# Characterization of *CXE* genes in pineapple and their aroma-related expression during fruit ripening

**DOI:** 10.3389/fpls.2025.1733743

**Published:** 2025-12-08

**Authors:** Wenxin Xu, Tangxiu Li, Jing Wu, You Wang, Junting Feng, Aiping Luan, Okandze Poho Pérol Carzorel, Shuqiang He, Junhu He, Chengjie Chen, Wuqiang Ma

**Affiliations:** 1 Sanya Institute of Breeding and Multiplication & Key Laboratory of Quality Regulation of Tropical Horticultural Crop in Hainan Province, School of Tropical Agriculture and Forestry, Hainan University, Sanya, Hainan, China; 2 State Key Laboratory of Tropical Crop Breeding, Key Laboratory of Crop Gene Resources and Germplasm Enhancement in South China, Ministry of Agriculture and Rural Affairs, Haikou, Hainan, China; 3 Key Laboratory of Tropical Crops Germplasm Resources Genetic Improvement and Innovation of Hainan Province, Tropical Crops Genetic Resources Institute, Chinese Academy of Tropical Agricultural Sciences, Haikou, Hainan, China; 4 Haikou Experimental Station Chinese Academy of Tropical Agricultural Sciences, Chinese Academy of Tropical Agricultural Sciences, Haikou, Hainan, China; 5 State Key Laboratory of Tropical Crop Breeding, Sanya Research Institute, Institute of Tropical Bioscience and Biotechnology, Chinese Academy of Tropical Agricultural Sciences, Sanya, Hainan, China; 6 National Centre for Crop Disease Control, Ministry of Agriculture, Animal Husbandry and Fisheries, Brazzaville, Republic of Congo

**Keywords:** pineapple, *CXE* gene family, carboxylesterase, esters, aroma

## Abstract

Carboxylesterases (CXEs) are ester hydrolyzing enzymes closely associated with the degradation of volatile esters and aroma release in fruit. Pineapple possesses a strong, ester-rich aroma, yet its *CXE* gene family has not been characterized. Here, we performed a genome-wide identification and analysis of the pineapple *CXE* family, detected 20 members, and pinpointed *AcCXE4* and *AcCXE7* as key negative regulators of aroma accumulation and prime candidates for aroma-oriented breeding. Phylogenetic comparison with *CXEs* from *Arabidopsis*, tomato, strawberry, Nanguo pear, and peach resolved five clades, in which *AcCXE4* grouped with *PuCXE15*, a reported aroma-related gene in Nanguo pear, while *AcCXE7* clustered with tomato *SlCXE1* and *AcCXE3* with apple *MdCXE1*, suggesting these members participate in ester metabolism in pineapple and are potential contributors to fruit-aroma formation. Besides, although *AcCXE13* and *AcCXE20* show conserved gene structure and sequence, their exon numbers and motif architectures differ from other *AcCXEs*, implying functional specialization. Comparative genomics indicated that family expansion in pineapple was driven primarily by tandem duplication and large segmental duplication. Integrating cis-regulatory element profiling, transcriptome analyses, and experimental validation, we found that most *AcCXEs* are likely responsive to light and hormone signaling (including the jasmonate pathway) and to abiotic stress cues. Several *AcCXE* genes exhibited decreasing expression across cultivars, tissues, and developmental stages, showing negative correlations with aroma accumulation, with *AcCXE4* and *AcCXE7* displaying the strongest association with pineapple aroma formation. Collectively, this work systematically defines the pineapple *CXE* family and highlights priority targets to inform molecular improvement of fruit aroma.

## Introduction

Pineapple (*Ananas comosus* (L.) Merr.) is one of the three major tropical fruits worldwide and is renowned for its distinctive, attractive aroma (Ali et al., 2020). In production, cultivar differences, fruit maturity, climate variability, cultivation practices (Coelho et al., 2024), and biotic stresses (Larrea-Sarmiento et al., 2022) directly influence the intensity and composition of pineapple fruit aroma, thereby affecting market quality. Over the past decade, studies have shown that during ripening, a substantial accumulation of esters, driven by accelerated biosynthesis and reduced hydrolysis, underpins pineapple’s characteristic flavor profile (Montero‐Calderón et al., 2010; Steingass et al., 2015; George et al., 2023, 2024).

The accumulation of ester aroma compounds in fruit is governed by two opposing processes, biosynthesis and degradation, with the latter largely mediated by carboxylesterases (CXEs). *CXEs* belong to the α/β-hydrolase superfamily, are widespread in plants, animals, and microbes, and are characterized by a conserved catalytic triad and a GXSXG motif (Kim et al., 1997), features that confer hydrolytic activity toward diverse ester substrates (Martínez-Rivas et al., 2022). In plants, the *CXE* family typically comprises on the order of a dozen to several dozen members, with copy number shaped by species ploidy and lineage-specific diversification. For example, 20 *CXEs* have been identified in *Arabidopsis* (Marshall et al., 2003), 33 in peach (Cao et al., 2019a), and 35 in grapevine (Zhang et al., 2022). Studies in Nanguo pear, apple, and peach have demonstrated that *CXEs* influence fruit-aroma formation by promoting ester degradation, exhibiting functional diversification and substrate specificity across taxa (Souleyre et al., 2011; Cao et al., 2019b; Qi et al., 2023). Recent advances in other economically important fruits have further expanded the understanding of CXEs-related aroma metabolism. In mango, revealed that rapid ripening involves dynamic transformations of aldehydes and esters driven by the catabolism of linoleic and linolenic acids, highlighting the crucial role of fatty acid-derived substrates in volatile formation (Wang et al., 2025). Similarly, studies in citrus have shown that CXEs-like and other hydrolase genes are associated with the modulation of terpenoid composition during fruit maturation, suggesting that esterases may also participate indirectly in the remodeling of terpene-derived volatiles (Hu et al., 2024). These findings underscore that CXE-mediated volatile metabolism represents a conserved yet functionally diversified mechanism among fruit species, providing a valuable reference for elucidating the aroma formation process in pineapple.

Despite esters being the predominant constituents of pineapple aroma, the *CXE* family underlying ester metabolism in pineapple has not been systematically characterized. Using the latest pineapple reference genome and annotations (Feng et al., 2024), we conducted a comprehensive analysis of the *CXE* gene family, including genome-wide identification, phylogenetic and duplication-origin analyses, sequence characterization, and expression profiling across germplasm with contrasting aroma intensity/composition and across fruit ripening stages, complemented by preliminary experimental validation. These results provide mechanistic insight into the molecular basis of pineapple aroma and establish foundational resources for its genetic improvement.

## Materials and methods

### Materials

Fruits of ‘Hongmi’ (HM), ‘Xiangshui’ (XS), ‘Mangguo’ (MG), ‘Weiduoliya’ (WD), and ‘HongXiangshui’ (HXS) were harvested from the pineapple germplasm orchard of the Tropical Crops Genetic Resources Research Institute, Chinese Academy of Tropical Agricultural Sciences, located at Baodao Xincun, Danzhou, Hainan, China (19°29′17″N, 109°29′4″E; elevation 130 m; mean annual temperature 23 °C; relative humidity 85%; annual precipitation ~1,500 mm). Fruits were sampled at three aroma development stages: the non-aromatic stage (approximately 56 days after flowering, with no detectable aroma by sensory evaluation and a total soluble solids (TSS) content of about 12–13°Brix), the initial aroma stage (approximately 63 days after flowering, with a faint fruity aroma detected and a TSS content of about 14–15°Brix), and the strong aroma stage (approximately 68 days after flowering, characterized by a pronounced sweet aroma and a TSS content of about 15–18°Brix). The stage classification was based on sensory evaluation in combination with days after flowering and total soluble solids content. After harvest, fruits were held in the laboratory for 24 h to equilibrate volatiles and minimize field temperature/humidity effects. Samples were then immediately frozen in liquid nitrogen and stored at -80 °C until analysis.

### Physicochemical characteristics of *AcCXE* family

The pineapple reference genome sequences and gene structural annotation were downloaded from the pineapple genome database (https://ananas.watchbio.cn). CXE protein sequences from *Arabidopsis* retrieved from UniProt were used as queries for BLASTP (v2.16.0) searches against the pineapple proteome (E-value < 1 × 10^-5^). Candidate AcCXEs were further screened by homology against the UniProtKB/Swiss-Prot database to remove redundant entries. Conserved domains were predicted with InterPro (https://www.ebi.ac.uk/interpro/, v107.0), and proteins containing the α/β-hydrolase fold (Pfam: PF07859) were retained as CXE candidates. All gene structures of *AcCXEs* were further curated by GSAman (https://tbtools.cowtransfer.com/s/a11146181df14f, v0.9.53). Physicochemical properties of AcCXE proteins were computed using TBtools-II (v2.363) (Chen et al., 2023), and subcellular localizations were predicted with WoLF PSORT (https://wolfpsort.hgc.jp/).

### Phylogenetic analysis of the *AcCXE* family

CXE protein sequences from Arabidopsis, peach, Nanguo pear, tomato, and apple were retrieved from NCBI, GDR (https://www.rosaceae.org/), and TAIR. Homologs were identified using two approaches hmmsearch and BLASTP (v2.16.0), and redundant entries were removed. The filtered CXE sets from these species were combined with pineapple AcCXEs to infer a maximum-likelihood phylogeny using the “One Step Build a ML Tree” tool in TBtools-II (v2.363). The resulting tree was formatted and annotated in Evolview (https://www.evolgenius.info/evolview/#/treeview).

### Chromosomal distribution and synteny analysis of the *AcCXE* family

*AcCXE* loci and their annotations were processed in TBtools-II (v2.363) (“One Step MCScanX—Super Fast”) to generate chromosome-level gene-distribution files and map the physical positions of *AcCXE* genes. Intra-genomic duplication relationships (tandem and segmental) among *CXEs* were then identified with MCScanX (v1.0.0). The results were visualized using the “Advanced Circos” module in TBtools-II (v2.363).

### Gene structure and conserved domain analysis of the *AcCXE* family

For gene structure and conserved-domain analyses, conserved domains of AcCXE proteins were predicted using NCBI CDD (Batch) (Marchler-Bauer and Bryant, 2004) and Pfam with default parameters. Conserved motifs were identified across the 20 AcCXE proteins using MEME Suite (https://meme-suite.org/meme/, v5.5.8) (Bailey et al., 2009), with the maximum number of motifs set to 10 to capture motif types and counts across subfamilies. Gene structure, conserved motifs, conserved domains, and sequence identifiers were then integrated and visualized in TBtools-II (v2.363).

### Promoter cis-acting element analysis of the *AcCXE* gene family in pineapple

Genomic coordinates of CXE loci were obtained from the GP genome GFF using TBtools-II (v2.363), and the 2,000-bp sequences upstream of the translation start codon (ATG) were extracted as putative promoter regions. These sequences were submitted to PlantCARE (http://bioinformatics.psb.ugent.be/webtools/plantcare/html/) for prediction of cis-acting regulatory elements (Lescot et al., 2002). Detected element types were then enumerated, classified, and summarized.

### Expression patterns of the *AcCXE* gene family across cultivars and fruit developmental stages in pineapple

Transcriptome datasets for root, stem, leaf, petal, ovule, and fruit core were obtained from NCBI BioProject PRJNA483249 (Mao et al., 2018). RNA-seq data for different cultivars and developmental stages were generated by our laboratory; three biological replicates per cultivar were sequenced, and expression values were averaged. All reads were quantified with Kallisto (v0.51.1) and normalized as TPM (Bray et al., 2016). Fruits of ‘Hongmi’ (HM), ‘Xiangshui’ (XS), and ‘HongXiangshui’ (HXS) were sampled at three aroma stages (aroma-absent, aroma-onset, aroma-intense). Total RNA was extracted and reverse-transcribed for RT–qPCR. Primer specificity was assessed using Primer5, and primers were synthesized by Wuhan Zhuandao Biotechnology Co., Ltd. Actin served as the reference gene (Yi et al., 2023; Zhang et al., 2024b). Relative expression was calculated with the 2^−ΔΔCt^ method (Rao et al., 2013), with three technical replicates per sample. Statistical analyses were performed in IBM SPSS Statistics 27 using two-tailed t-tests, and figures were prepared in GraphPad Prism 8.0.

## Results

### Characteristics of *AcCXE* family members in pineapple

From the pineapple genome, we identified 20 *CXE* family members and named them *AcCXE1*–*AcCXE20* in ascending order of chromosomal position. Protein physicochemical analysis showed lengths of 169–463 amino acids and predicted isoelectric points (pI) of 4.73–8.93 (**Table 1**). Subcellular localization prediction indicated 12 proteins in the cytosol and 6 in chloroplasts; AcCXE10 was predicted to localize to the endoplasmic reticulum, and AcCXE9 to the nucleus. 

**Table 1:** Members of the *AcCXE* gene family in pineapple (*Ananas comosus*) and their physicochemical properties.

Gene ID	Rename ID	Number of amino acid	Molecular weight	Theoretical pI	Instability index	Aliphatic index	Grand average of hydropathicity	Predicted location(s)
lcfv2_02654.t1	*AcCXE1*	319	34994.48	5.27	36.61	78.09	-0.24	Cytoplasm
lcfv2_02655.t1	*AcCXE2*	310	33256.95	5.46	42.08	88.61	-0.034	Cytoplasm
lcfv2_04648.t1	*AcCXE3*	334	36681.01	6.81	65.84	64.64	-0.517	Chloroplast
lcfv2_05640.t1	*AcCXE4*	372	41286.28	6.35	45.97	79.73	-0.331	Cytoplasm
lcfv2_05661.t1	*AcCXE5*	355	38085.51	5.51	55.88	93.49	-0.009	Cytoplasm
lcfv2_06897.t1	*AcCXE6*	463	49249.31	8.93	55.75	86.05	-0.038	Chloroplast
lcfv2_08066.t1	*AcCXE7*	330	35655.03	5.61	33.33	80.52	-0.238	Chloroplast
lcfv2_08068.t1	*AcCXE8*	379	40466.92	8.42	49.46	77.28	-0.078	Chloroplast
lcfv2_09598.t1	*AcCXE9*	343	37921.94	5.36	51.86	77.9	-0.25	Nucleus
lcfv2_09599.t1	*AcCXE10*	335	36996.01	5.49	51.31	82.72	-0.173	Endoplasmic Reticulum
lcfv2_09600.t1	*AcCXE11*	334	36752.81	5.62	51.21	86.41	-0.149	Cytoplasm
lcfv2_09601.t1	*AcCXE12*	336	36898.65	5.27	50.26	83.01	-0.183	Cytoplasm
lcfv2_10044.t1	*AcCXE13*	317	33841.25	4.97	39.95	89.65	-0.101	Cytoplasm
lcfv2_11631.t1	*AcCXE14*	332	36046.79	5.34	57.79	80.54	-0.147	Cytoplasm
lcfv2_18586.t1	*AcCXE15*	334	36419.7	8.61	34.01	88.23	-0.001	Cytoplasm
lcfv2_20062.t1	*AcCXE16*	454	48005.03	8.67	48.47	88.68	0.03	Chloroplast
lcfv2_22485.t1	*AcCXE17*	335	36282.22	6.35	46.94	82.48	-0.07	Cytoplasm
lcfv2_22564.t1	*AcCXE18*	327	36232.97	5.42	55.14	86.27	-0.351	Cytoplasm
lcfv2_22565.t1	*AcCXE19*	330	36375.12	4.92	39.93	85.42	-0.302	Chloroplast
lcfv2_24465.t1	*AcCXE20*	340	36661.36	4.73	51.68	86	-0.039	Cytoplasm

Predicted subcellular localization was determined using WoLF PSORT (https://wolfpsort.hgc.jp/).

### Phylogenetic and evolutionary analysis of the *AcCXE* family

**Figure 1:**
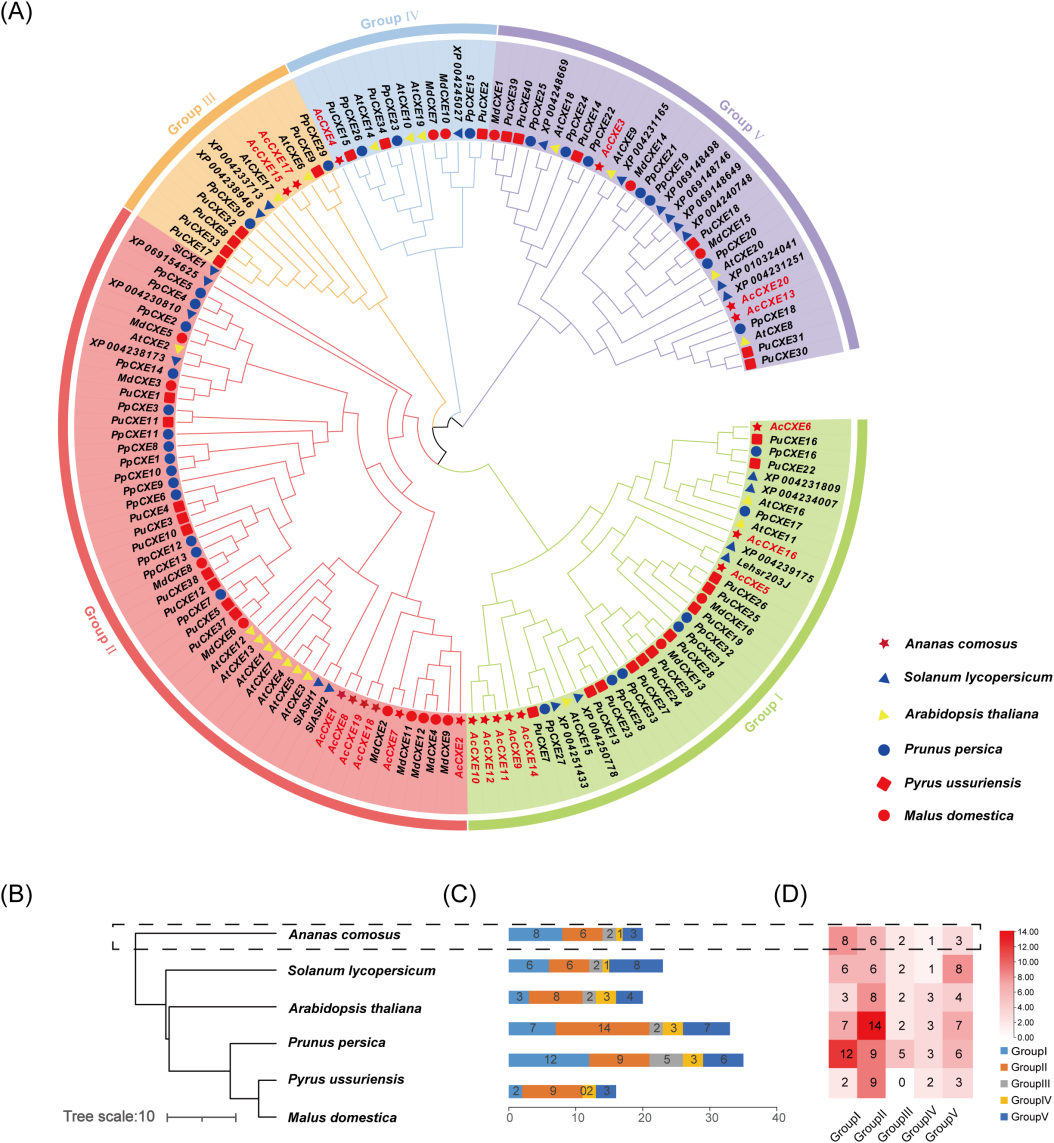
Maximum-likelihood phylogeny of CXE proteins from six species. **(A)** Phylogenetic relationships of *CXE* gene families across six species, resolving five clades. **(B)** Species tree of the six taxa from TimeTree. **(C–D)** Proportional composition of CXE clades within each species. *Ananas comosus* (red star), *Arabidopsis thaliana* (yellow triangle), *Pyrus ussuriensis* (red square), *Prunus persica* (blue circle), *Solanum lycopersicum* (blue triangle), *Malus domestica* (red circle).

To elucidate the evolution of the pineapple CXE family, we constructed a maximum-likelihood (ML) phylogeny comprising 147 CXE proteins from six species: 20 from pineapple, 20 from *Arabidopsis thaliana*, 35 from Nanguo pear (*Pyrus ussuriensis*), 33 from peach (*Prunus persica*), 23 from tomato (*Solanum lycopersicum*), and 16 from apple (*Malus domestica*), and grouped them accordingly (**Figure 1A**). The tree resolved five major clades (Group I–V), with Group I and Group II containing the largest numbers of members (**Figure 1D**), suggesting these clades dominated family expansion. Pineapple CXEs were distributed across all five clades. Notably, AcCXE4 clustered with PuCXE15, a gene implicated in ester degradation in Nanguo pear (Qi et al., 2023); AcCXE7 clustered with tomato SlCXE1 (Goulet et al., 2012), and AcCXE3 with apple MdCXE1 (Souleyre et al., 2011), indicating that AcCXE4, AcCXE7, and AcCXE3 may participate in ester hydrolysis in pineapple. In terms of family size, Nanguo pear and peach harbored the most CXEs (35 and 33, respectively), whereas pineapple possessed 20. Overall, the phylogeny and gene counts indicate clade-level conservation with lineage-specific diversification.

### Chromosome distribution and synteny analysis of the *AcCXE* family

**Figure 2:**
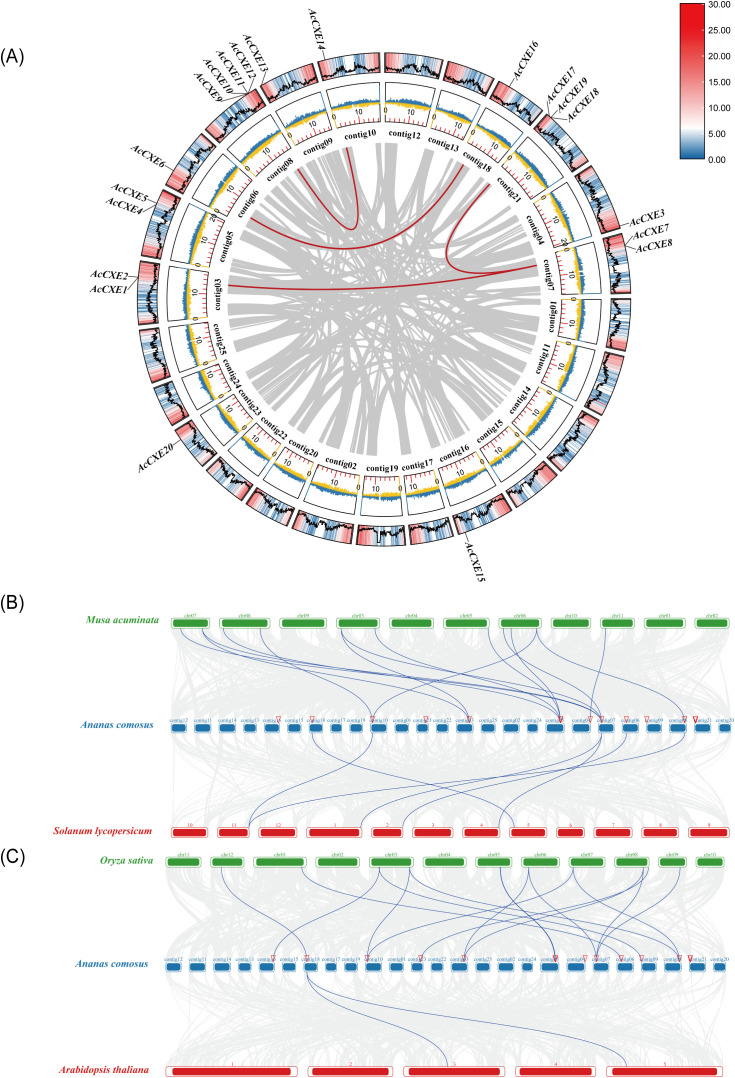
Comparative genomic analysis between pineapple and other species, focusing on *CXE* gene family. **(A)** Intragenomic synteny among *AcCXE* loci. Circos tracks (from inner to outer): GC skew density, gene density, and GC content. **(B)** Inter-species synteny between pineapple and banana/*Solanum lycopersicum* (tomato). **(C)** Inter-species synteny between pineapple and *Oryza sativa* (rice)/ *Arabidopsis thaliana*.

To assess the chromosome distribution of *AcCXEs*, we visualized their loci with a Circos plot (**Figure 2A**). The genes are dispersed across 12 chromosomes. Single-copy loci occur on contig04, contig06, contig10, contig16, contig18, and contig23, indicating an overall scattered pattern. Several genes co-localize on the same chromosome, for example, *AcCXE1* and *AcCXE2* on contig03, and *AcCXE17* and *AcCXE19* on contig21. Inspection of collinearity links revealed both tandem and segmental duplications that likely contributed to family expansion: *AcCXE7*-*AcCXE8*-*AcCXE4*-*AcCXE5* form a tandem array, whereas *AcCXE6* and *AcCXE16* represent a segmental duplicate pair.

Comparative synteny showed conserved collinearity between pineapple *CXE* loci and those in banana, tomato, rice, and *Arabidopsis*, albeit with different counts. We detected 14 and 6 syntenic pairs between pineapple and banana or tomato, respectively (**Figure 2B**), and 15 and 2 pairs between pineapple and rice or *Arabidopsis*, respectively (**Figure 2C**). The higher numbers for banana and rice relative to tomato and *Arabidopsis* suggest substantial divergence in the chromosomal neighborhoods harboring *CXE* genes between monocots and dicots. Given that banana and tomato are well-studied for fruit aroma, these syntenies provide a useful reference for inferring the roles of pineapple CXEs in volatile ester metabolism and aroma formation.

### Conserved motifs, domains and gene structure analysis of AcCXEs

**Figure 3:**
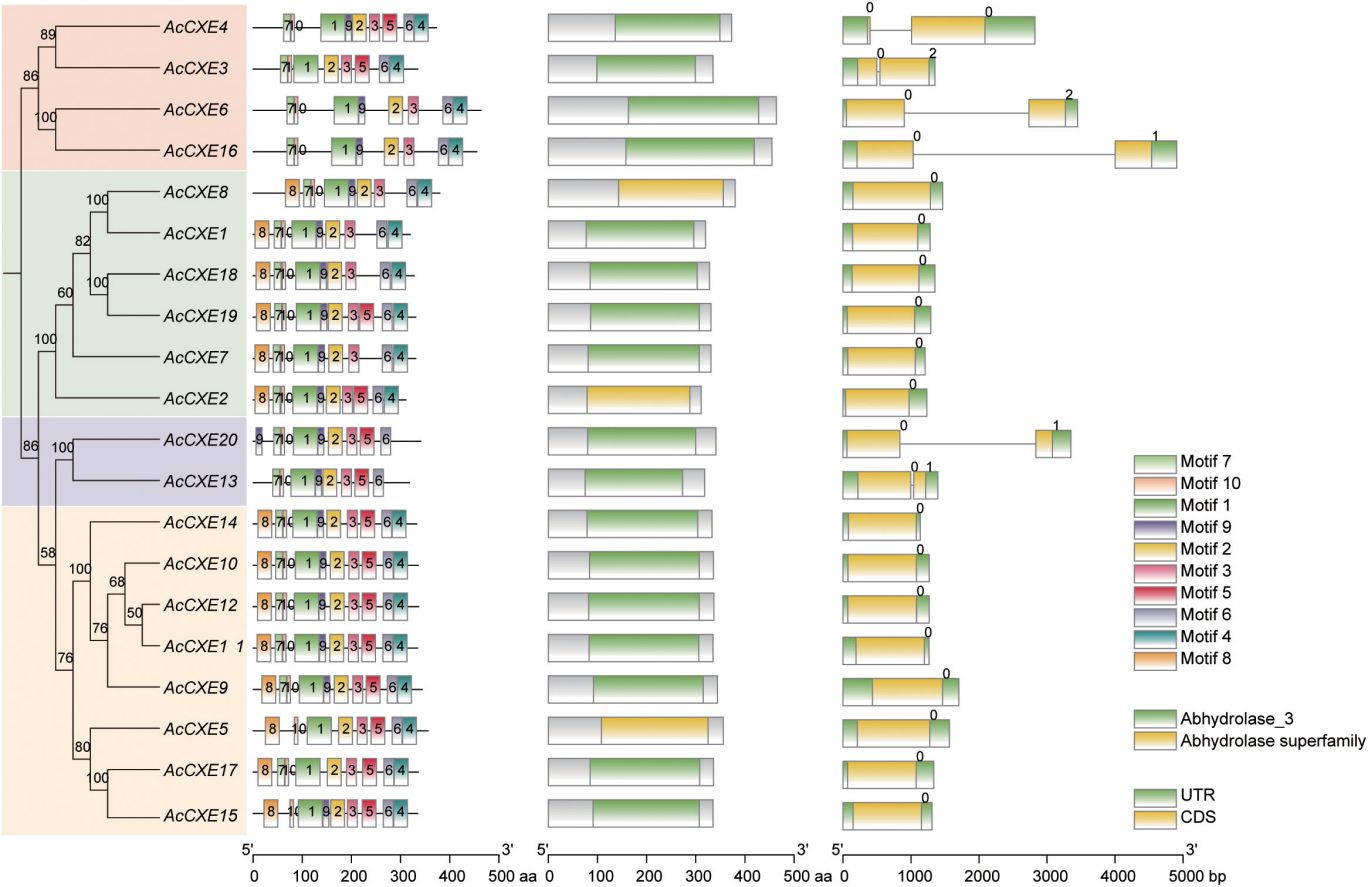
Conserved features analysis of the *AcCXE* gene family. From left to right, phylogenetic relationships, conserved motifs, functional domains, and gene structure of *AcCXEs*, respectively.

To further explore conserved features of pineapple *CXEs*, we analyzed their protein domains and motifs. Ten conserved motifs were identified across *AcCXE* sequences (**Figure 3B**). Motifs 1, 2, 3, 6, and 10 are highly conserved and present in all members. Motif number and order are broadly consistent within subclades, whereas several genes carry fewer motifs-e.g., *AcCXE15*-suggesting functional specialization. Gene-structure analysis (**Figure 3D**) showed that most *AcCXEs* contain 3–4 exons; members within the same phylogenetic branch share similar architectures (**Figure 3A**), indicating lineage-specific structural diversification. AcCXE13 and AcCXE20 are conserved at the gene-structure and sequence levels, yet their exon counts and motif compositions differ from most other *AcCXEs*, implying possible neofunctionalization. All AcCXEs harbor the conserved α/β-hydrolase superfamily domain, and most also contain the Abhydrolase_3 domain.

### Promoter cis-acting element analysis of the *AcCXE* gene family in pineapple

**Figure 4:**
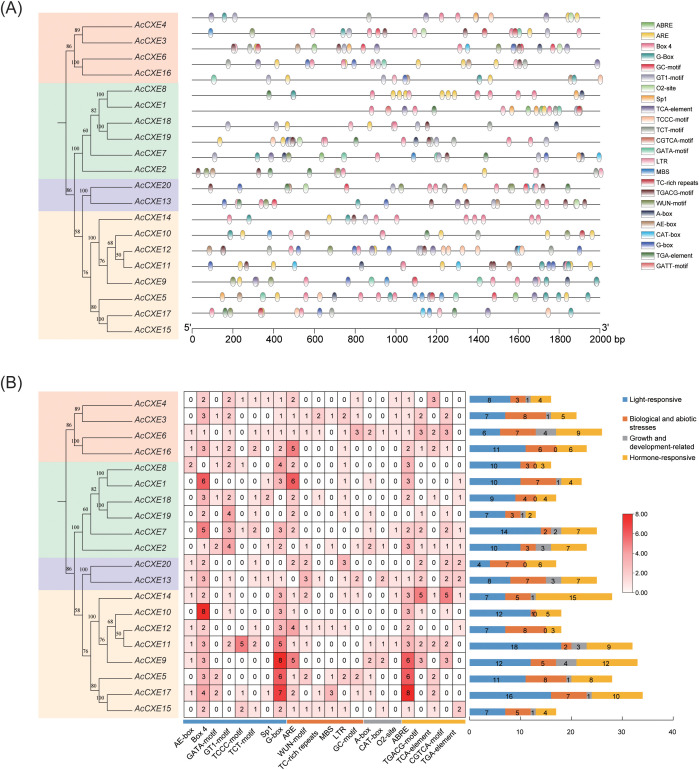
Cis-element analysis of *AcCXEs*’ promoters. **(A)** Chromosome distributions of different classes of cis-elements in pineapple *CXE* gene promoter sequences; **(B)** Statistics analysis of cis-elements of *AcCXEs*’ promoter sequences.

To explore potential regulatory events of *AcCXE* genes in abiotic stress and development, we surveyed cis-acting elements within the 2,000-bp upstream promoter regions (**Figure 4**). After excluding ubiquitous core elements such as the CAAT-box and TATA-box, a total of 452 cis-elements were identified and classified into four major categories: light-responsive, stress-responsive (abiotic), development-related, and hormone-responsive elements.

Among them, light-responsive cis-elements were abundant—including G-box, Box4, GT1-motif, and TCT-motif. Box4 occurred most frequently in *AcCXE10*, whereas G-box was most frequent in *AcCXE9*, suggesting important roles for these genes in light signal transduction. Hormone-responsive elements were enriched for ABRE (ABA-responsive), TGACG-motif (MeJA-responsive), TGA-element (auxin-responsive), and TCA-element (salicylic acid–responsive); the prevalence of MeJA-related motifs indicates extensive involvement of the family in jasmonate-mediated regulation. Stress-associated elements (ARE, MBS, LTR, TC-rich repeats) were common, implicating *AcCXEs* in responses to low temperature, drought, and anaerobic stress. Development-related elements—including CAT-box, A-box, and O2-site—were also frequent, consistent with regulation of tissue-specific expression and developmental processes.

### Expression pattern analysis of the *AcCXE* gene family across cultivars and developmental stages in pineapple

**Figure 5:**
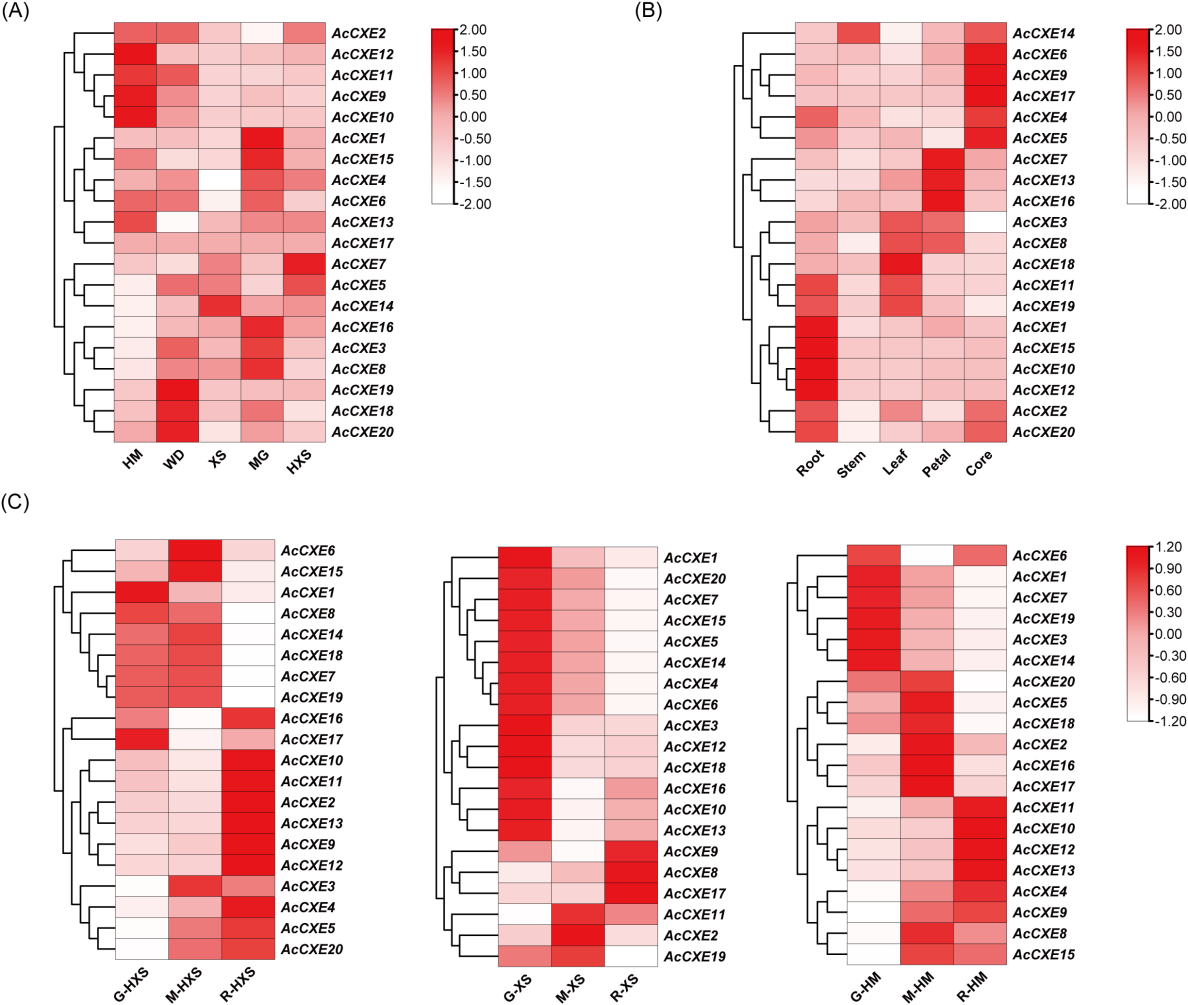
Expression profiles of *AcCXEs* across different varieties, tissues and fruit development stages. **(A)** Transcript abundance of *AcCXE* genes across cultivars. **(B)** Transcript abundance across tissues. **(C)** Transcript abundance across aroma-development stages in three aroma-type cultivars (HM, XS, HXS). All expression data are estimated using TPM method, generated from RNA-seq experiments, averaged over three biological replicates.

To characterize the expression profile of *AcCXEs* across cultivars, tissues, and fruit development, we analyzed RNA-seq datasets. By cultivar (**Figure 5A**), *AcCXE5* and *AcCXE7* were highly expressed in the light-aroma cultivar ‘HongXiangshui’ (HXS), consistent with a putative negative role in aroma formation. By tissue (**Figure 5B**), *AcCXE4*, *AcCXE5*, and *AcCXE9* showed elevated expression in the fruit core, which exhibits weak aroma, again aligning with negative regulation. Across developmental stages (**Figure 5C**), *AcCXE3*, *AcCXE4*, and *AcCXE13* were down-regulated as fruit aroma intensified, suggesting repression of ester accumulation. Collectively, these patterns nominate six candidates, *AcCXE3*, *AcCXE4*, *AcCXE5*, *AcCXE7*, *AcCXE9*, and *AcCXE13*, as key genes associated with pineapple aroma metabolism.

### RT–qPCR validation of *AcCXE* gene expression in pineapple

**Figure 6:**
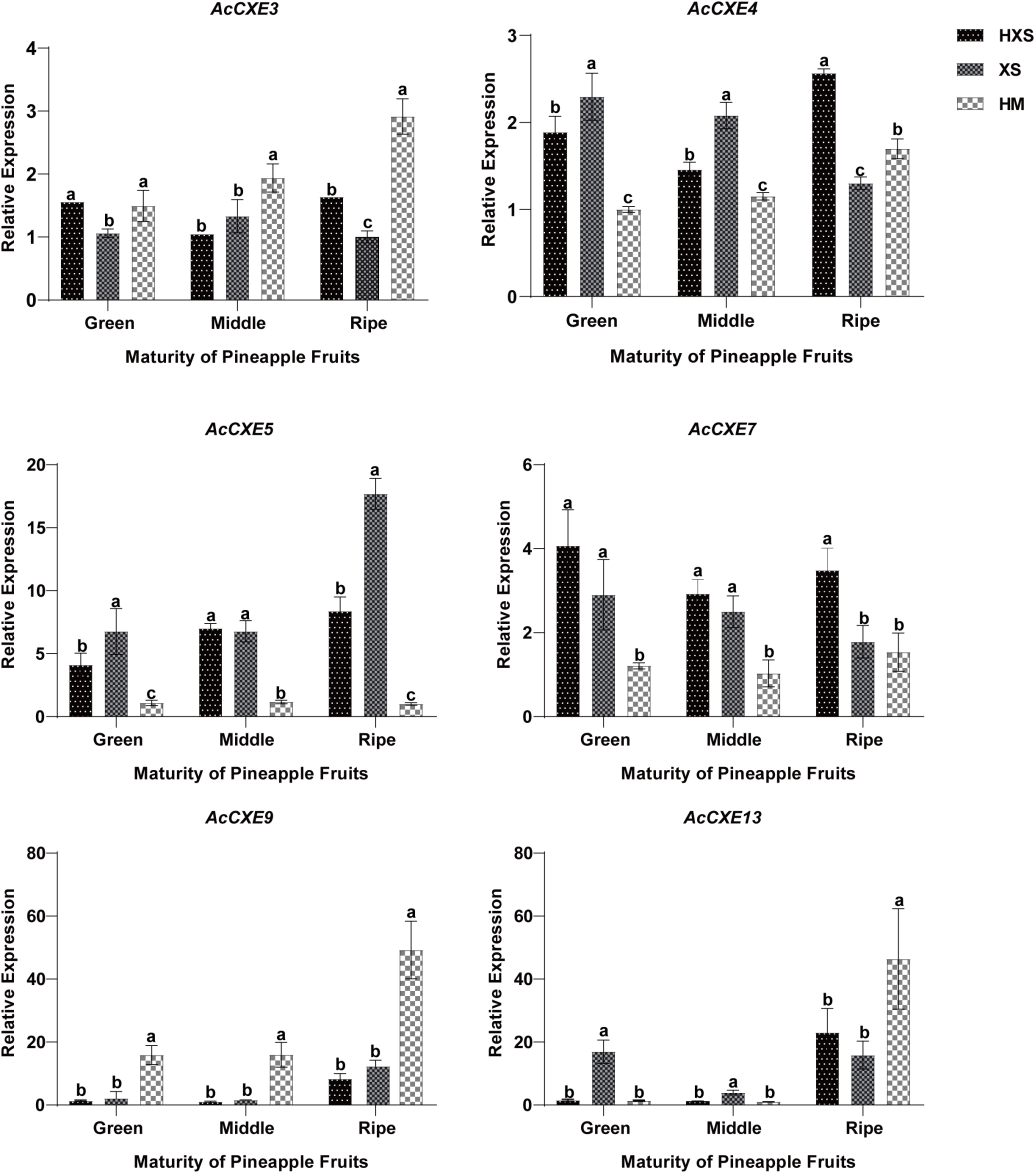
Relative qRT-PCR expression of six *AcCXE* genes across ripening stages in three pineapple cultivars. Expression was normalized to Actin and calculated by the 2^−ΔΔCt^ method; values are means ± SD (n = 3). HXS, ‘Hong Xiangshui’; XS, ‘Xiangshui’; WD, ‘Victoria’; MG, ‘Mango’; HM, ‘Hongmi’. Ripening stages: Green (immature, green peel), Middle (partially ripe), Ripe (fully ripe). Different lowercase letters indicate significant differences among groups (P < 0.05 or P < 0.01).

Six candidates, *AcCXE3*, *AcCXE4*, *AcCXE5*, *AcCXE7*, *AcCXE9*, and *AcCXE13*, were selected for RT-qPCR validation (**Figure 6**). *AcCXE4* and *AcCXE7* showed higher expression in the light-aroma cultivar ‘HongXiangshui’ (HXS) than in the sweet/fruit-aroma cultivars ‘Hongmi’ (HM) and ‘Xiangshui’ (XS). Notably, *AcCXE4* expression decreased with ripening in XS but increased in HXS, consistent with a role for *CXEs* as negative regulators of ester accumulation. These results implicate *AcCXE4* and *AcCXE7* as key genes closely associated with pineapple aroma formation.

## Discussion

We comprehensively characterized the pineapple *AcCXE* family by assessing copy number and physicochemical properties, phylogeny, gene structure, conserved motifs, promoter cis-elements, and expression patterns, and we conducted RT-qPCR for preliminary validation. The 20 *AcCXE* members identified in the genome differ in basic features, with protein lengths of 170–460 amino acids and predicted isoelectric points of 4.7-8.9, indicating potential structural and functional divergence. Subcellular localization predictions placed most CXEs in the cytosol, with a minority in chloroplasts, the endoplasmic reticulum, and the nucleus, suggesting a predominant role in cytosolic ester hydrolysis.

Phylogenetically, pineapple CXEs clustered with CXEs from model plants (for example, *Arabidopsis thaliana*, *Solanum lycopersicum*, and strawberry) and fruit trees (for example, Nanguo pear) into five clades, supporting broad evolutionary conservation together with lineage-specific expansions in certain groups (for example, Group IV) (Goulet et al., 2012; Rui et al., 2022; Zhang et al., 2024a). Several *AcCXEs* grouped with genes previously implicated in fruit-aroma regulation: *AcCXE4* with *PuCXE15*, *AcCXE7* with tomato *SlCXE1*, and *AcCXE3* with apple *MdCXE1*. Given that *PuCXE15* promotes ester degradation in Nanguo pear (Qi et al., 2023) and that *MdCXE1* and *SlCXE1* mediate ester catabolism in apple and tomato (Souleyre et al., 2011; Goulet et al., 2012), the corresponding pineapple genes likely participate in volatile ester turnover during pineapple aroma formation.

In gene structure and motif analyses, *AcCXEs* showed an overall conserved organization, which helps resolve relationships among family members (Babenko et al., 2004; Roy and Penny, 2007). Exon number and length varied among genes. Such shifts, often coupled with exon count and length, are characteristic of gene structural evolution (Zhu et al., 2009). Domain analysis indicated that most AcCXEs contain the conserved carboxylesterase domain. *AcCXE13* lacks Motif 4 and Motif 8, both of which are conserved in most *AcCXE* members. Motif 4 is generally located near the catalytic serine residue and is considered critical for forming the catalytic pocket and recognizing ester substrates, whereas Motif 8 contributes to maintaining the stability of the α/β-hydrolase fold. The absence of these motifs in *AcCXE13* may therefore lead to structural alteration or loss of catalytic efficiency, implying potential functional divergence from other AcCXE proteins. Motif variation may underlie functional divergence, and structural diversity likely confers substrate specificity and regulatory flexibility within the *AcCXE* family (Su et al., 2020).

*AcCXEs* were distributed across all chromosomes, with clustered arrays in several regions. Tandem and segmental duplications are likely the principal drivers of family expansion (Die et al., 2018). Comparative synteny with tomato, rice, *Arabidopsis*, and banana revealed 14 and 15 syntenic pairs with the monocots, banana and rice, and 6 and 2 pairs with the dicots tomato and *Arabidopsis*. This pattern indicates stronger conservation of CXE loci in monocots and suggests functional diversification after the monocot-dicot divergence. Building on established findings for fruit aroma in banana and tomato, these collinear relationships support roles for pineapple *CXEs* in ester metabolism and aroma formation.

Prediction of cis-acting elements indicated that *AcCXEs* may respond to light, hormones, abiotic stress, and developmental cues. Light-responsive elements were most abundant. Box4 was most frequent in *AcCXE10* and G-box was most frequent in *AcCXE9*, suggesting roles in light signaling and possible involvement in ester metabolism (Ain-Ali et al., 2021). Among hormone-related elements, jasmonate-responsive motifs were most enriched, indicating participation of the *AcCXE* family in the JA pathway, which is important for fruit ripening and defense responses (Wasternack and Hause, 2013). Stress-associated elements such as ARE, MBS, and LTR were enriched, suggesting that some *AcCXEs* respond to low temperature, drought, and anaerobic stress. Furthermore, the distribution of hormone-related elements may help explain the differential expression of certain *AcCXEs* genes among pineapple varieties. For instance, *AcCXE4* and *AcCXE7* showed higher expression in the low-aroma variety ‘HongXiangshui’, which may be associated with the abundance of ABA-responsive elements (ABRE) in their promoter regions. These elements might be activated during the late maturation stage of this variety, thereby enhancing gene expression and accelerating ester degradation, ultimately contributing to its weaker aroma intensity.

Integrating RNA-seq and qPCR, we systematically screened *AcCXE* genes potentially associated with aroma intensity. At the transcript level, *AcCXE3*, *AcCXE4*, *AcCXE5*, *AcCXE7*, *AcCXE9*, and *AcCXE13* showed expression patterns consistent with the negative regulation of ester accumulation by CXEs. qPCR across cultivars showed that *AcCXE4* and *AcCXE7* were expressed at significantly higher levels at ripening in the light-aroma cultivar ‘HongXiangshui’ than in the sweet-aroma cultivars. Their expression decreased with ripening in ‘Xiangshui’ but increased in ‘HongXiangshui’, which may underlie cultivar differences in aroma content. The expression of *PuCXE15*, which clustered together with *AcCXE4*, is highly negatively correlated with the contents of key esters, and its overexpression or silencing, respectively, leads to a decrease or increase in ester content, suggesting that this gene plays a direct role in aroma regulation. We therefore infer that *AcCXE4* and *AcCXE7* modulate ester metabolism to influence volatile-aroma production, with cultivar- and stage-dependent effects.

In summary, the pineapple *AcCXE* family constitutes a conserved, multifunctional regulatory network shaped by long-term gene duplication and functional divergence. AcCXEs likely participate in ester turnover, modulation of fruit aroma, regulation of stress responses, and control of development and growth. These findings provide a foundation for functional studies and molecular improvement. Future work can resolve AcCXE-mediated regulation of aroma biosynthesis and stress responses at the molecular and metabolomic levels.

## Conclusion

We systematically identified and analyzed 20 *CXE* genes in pineapple. Comprehensive assessments of physicochemical properties, phylogeny, gene structure, conserved motifs, chromosomal distribution, promoter cis-elements, expression profiles, and RT-qPCR highlighted *AcCXE4* and *AcCXE7*, whose expression patterns were negatively correlated with aroma formation, consistent with CXE-mediated ester catabolism. We infer that these genes likely mediate degradation of volatile esters and thereby shape pineapple aroma, making them priority targets for future aroma research and breeding.

## Data Availability

The original contributions presented in the study are included in the article/supplementary material. Further inquiries can be directed to the corresponding authors.
